# CUMULATIVE EFFECTS OF COGNITIVE IMPAIRMENT AND FRAILTY ON GERIATRIC SYNDROMES IN A GERIATRIC OUTPATIENT SETTING

**DOI:** 10.1093/geroni/igad104.2942

**Published:** 2023-12-21

## Abstract

**Aim:**

With underlying geriatric syndromes, only few studies emphasized on the aspect of co-occurrence of frailty and cognitive impairment, though they are independently associated with several geriatric syndromes.

**Methods:**

The participants were recruited from the outpatient clinic of geriatric department in a medical center. Frailty was assessed by a modified version of Fried’s criteria, while cognitive impairment was defined by cutoff scores of the Mini Mental State Examination. Demographic characteristics, self-assessed health status, and geriatric assessment were collected. Polytomous logistic regression model was used to evaluate factors correlated with frailty alone, cognitive impairment alone, and co-occurrence of frailty and cognitive impairment.

**Results:**

The mean age of the 323 participants (60.1% women) was 80.4 ± 7.3 years. The prevalence of non-frailty, frailty alone, cognitive impairment alone and co-occurrence were 34.4%, 16.7%, 23.8% and 25.1%, respectively. When compared to the not-frail group, the odds ratios (OR) of having activity of daily living impairment (aOR 1.44, 95% CI 1.28-1.63), instrumental activities of daily living impairment (aOR 1.61, 95% CI 1.45-1.81), urinary incontinence (aOR 1.61, 95% CI 1.45-1.81) and with lower mini-nutritional assessment score (aOR 148, 95% CI 1.30-1.69) were significantly higher in the co-occurrence group after adjusted with demographic variables and Charlson comorbidity index.

**Conclusions:**

Co-occurrence of frailty and cognitive impairment was associated with higher prevalence of disability, incontinence and malnutrition. It is crucial to approach frailty and cognitive impairment simultaneously in daily geriatric clinical practice due to the correlations of several geriatric syndromes to the co-occurrence of physical frailty and cognitive impairment.

